# Bifidobacteria Exhibit LuxS-Dependent Autoinducer 2 Activity and Biofilm Formation

**DOI:** 10.1371/journal.pone.0088260

**Published:** 2014-02-05

**Authors:** Zhongke Sun, Xiang He, Vincenzo F. Brancaccio, Jing Yuan, Christian U. Riedel

**Affiliations:** 1 Institute of Microbiology and Biotechnology, University of Ulm, Ulm, Germany; 2 Institute of Disease Control and Prevention, Academy of Military Medical Sciences, Beijing, China; The Scripps Research Institute and Sorrento Therapeutics, Inc., United States of America

## Abstract

Autoinducer-2 (AI-2) molecules are one class of signalling molecules involved in gene regulation dependent on population density in a mechanism commonly referred to as quorum sensing (QS). AI-2 is produced by the methylthioadenosine/S-adenosyl-homocysteine nucleosidase LuxS. In the present study, we characterise the function of bifidobacterial LuxS proteins to address the question whether these economically important bacteria are able to perform QS communication. All publically available genome sequences of bifidobacteria harbour putative *luxS* genes. The deduced amino acid sequences are well conserved in the genus and show good homology to the LuxS protein of the prototypical AI-2 producer *Vibrio harveyi*. The *luxS* genes of three bifidobacterial strains were successfully expressed in AI-2-negative *Escherichia coli* DH5α. Supernatants of these recombinant *E. coli* strains contained significant AI-2 activity. In initial experiments, we failed to detect AI-2 activity in supernatants of bifidobacteria grown in MRSc. High concentration of glucose as well as acidic pH had strong inhibitory effects on AI-2 activity. AI-2 activity could be detected when lower volumes of supernatants were used in the assay. Homologous overexpression of *luxS* in *Bifidobacterium longum* NCC2705 increased AI-2 levels in the supernatant. Furthermore, over-expression of *luxS* or supplementation with AI-2-containing supernatants enhanced biofilm formation of *B. longum* NCC2705. Collectively, these results suggest that bifidobacteria indeed harbour functional *luxS* genes that are involved in the production of AI-2-like molecules. To the best of our knowledge, this represents the first report on AI-2 activity produced by bifidobacteria. Self-produced AI-2 activity as well as AI-2-like molecules of other bacteria of the intestinal tract may have a regulatory function in biofilm formation and host colonization by bifidobacteria.

## Introduction

Quorum sensing (QS) is a mechanism for regulation of gene expression in response to the density of a bacterial population [1]. QS requires the production and detection of specific signalling molecules, which are commonly referred to as autoinducers [1]. Autoinducer-2 (AI-2)-like molecules are a class of signalling molecules that are a by-product of the activated methyl cycle for recycling of S-adenosylmethionine. In this process, the methylthioadenosine/S-adenosyl-homocysteine (MTA/SAH) nucleosidase LuxS converts S-ribosylhomocysteine to homocysteine and 4,5-dyhdroxy-2,3-pentanedione, which is the precursor of AI-2. Spontaneous cyclization of 4,5-dihdroxy-2,3-pentanedione results in the compound methyltetrahydroxyfuran, which in boron-rich environments leads to the spontaneous formation of a boron ester. Both methyltetrahydroxyfuran and its boron ester have AI-2 activity [2]. Some bacteria possess S-adenosylhomocysteine hydrolase as an alternative enzyme to LuxS, that converts S-adenosylhomocysteine to adenosine and homocysteine and hence do not produce AI-2 molecules [2].

LuxS-dependent AI-2 formation and its role in QS have been studied in detail in the Gram-negative, marine bacterium *Vibrio harveyi* and a number of other bacteria (reviewed in [3]). AI-2 production was proposed to serve not only as a QS mechanism within a bacterial species but also as a means for communication across the species barrier and possibly across different genera [4]. However, this hypothesis has been challenged by the fact that most bacteria that possess LuxS homologues do not encode for a LuxPQ two component system or the LsrB protein that serve as specific receptors for AI-2 molecules in *Vibrio sp.* or *Enterobacteriaceae*, respectively [5]. Other, specific AI-2 receptors have not been identified in bacteria that lack LuxPQ or LsrB. Nevertheless, most of the bacteria that possess LuxS but lack a specific AI-2 receptor show AI-2-dependent QS phenotypes, e.g. in biofilm formation or virulence [5].

In *V. harveyi*, bioluminescence is induced at high population densities in response to an increased concentration of AI-2 [6], [7]. More recently, AI-2 signalling has been shown to be involved in biofilm formation, virulence, production of antimicrobials, motility and genetic competence in a number of Gram-positive and Gram-negative bacteria [3], [8]. While production of AI-2-like molecules is not restricted to pathogens, AI-2 dependent QS phenotypes have been mostly studied in pathogenic microorganisms [3].

The assay employing *V. harveyi* BB170, a strain initially developed to identify the AI-2 molecule of *Vibrio sp.*, has become the method of choice to detect AI-2 activity in culture supernatants of bacteria [9]. The detection is based on the ability of *V. harveyi* BB170 to emit light by luminescence specifically in response to QS signals. Due to the absence of the sensors for other QS signals (i.e. autoinducer 1 or acetylhomoserine lactones), the *V. harveyi* strain BB170 specifically responds to AI-2-like molecules [6].

The only *Bifidobacterium sp.* strain for which a *luxS* homologue was reported so far is *B. longum* NCC2705 [10], [11]. The corresponding protein was recently detected in a 2D proteomic analysis of *B. longum* NCC2705 (supplementary Table S1 of Ref. [11]). Comparing the proteomes of this strain under *in vitro* and *in vivo* conditions, two isoforms of LuxS were detected with one being more abundant *in vivo* [12]. However, AI-2 activity and AI-2-dependent QS have neither been reported nor studied in this important group of microorganisms. Bifidobacteria are among the first colonizers of the gastrointestinal tract after birth and represent one of the dominant groups of normal human gastrointestinal microbiota [13], [14]. Beneficial effects on the host have been attributed to certain strains of bifidobacteria [15], [16] and some of these strains are marketed as probiotics. In this context, AI-2-dependent biofilm formation, e.g. on food particles or host-derived mucus, could be an important mechanism for early colonization of the host by commensal strains or persistence for prolonged periods by probiotic strains.

In order to investigate whether bifidobacteria exhibit LuxS-dependent AI-2 activity, the presence of LuxS homologues in the genus *Bifidobacterium* was analysed *in silico*. Three strains of bifidobacteria were selected to study the function of LuxS homologues in AI-2 production. Furthermore, biofilm assays were performed and provide a link between AI-2 production and biofilm formation of *B. longum* NCC2705, a well-studied probiotic candidate strain.

## Materials and Methods

### Bacterial strains, medium and growth conditions

All bacterial strains and plasmids used in this study are listed in Table 1. Bacterial strains were maintained at −80°C in standard media containing 20% glycerol. *E. coli* strains were routinely cultivated aerobically in LB medium at 37°C and *V. harveyi* BB170 was grown aerobically in AB medium at 28°C. Bifidobacteria were cultured in Lactobacilli MRS medium (BD Difco™) supplemented with 0.5 g/L L-cysteine (MRSc) at 37°C in sealed jars with anaerobic conditions generated using AnaeroGen™ (Oxoid). Bacterial growth was monitored by measuring optical density at 600 nm (OD_600_) using a spectrophotometer and the pH was measured in culture supernatants using a standard pH electrode. Growth of *V. harveyi* BB170 was assayed in transparent 96-well microtiter plates using an Infinite M200 multilabel plate reader (Tecan).

**Table 1:** Bacterial strains, plasmids and oligonucleotides used in this study.

Name	Relevant feature	Reference
**Strains**		
*E. coli* DH5α	Cloning host	Invitrogen
*E. coli* DH5α pBAD_LuxS_NCC_	Amp^R^, host for arabinose-inducible expression of *luxS* of *B. longum* NCC2705	this study
*E. coli* DH5α pBAD_LuxS_S17_	Amp^R^, host for arabinose-inducible expression of *luxS* of *B. bifidum* S17	this study
*E. coli* DH5α pBAD_LuxS_E18_	Amp^R^, host for arabinose-inducible expression of *luxS* of *B. longum* E18	this study
*E. coli* DH5α pBAD_LuxS_Vh_	Amp^R^, host for arabinose-inducible expression of *luxS* of *V. harveyi* BB170	this study
*B. longum* NCC2705	Sequenced type strain	[33]
*B. longum* NCC2705 pMgap	Spec^R^, pMgap, empty vector control	this study
*B. longum* NCC2705 pMgap_LuxS_NCC_	Spec^R^, vector for constitutive, homologous expression of LuxS of *B. longum* NCC2705	
*B. bifidum* S17	Faecal isolate of a breast-fed infant, genome sequenced	[34]
*B. longum* E18	Faecal isolate of an adult, genome sequenced	[35]
*V. harveyi* BB170	AI-2 reporter strain, *luxN*::Tn5	[36]
**Plasmids**		
pBAD	Amp^R^, vector for arabinose-inducible expression in *E. coli*	this study
pBAD_LuxS_E18_	Amp^R^, pBAD-based vector for arabinose-inducible expression of *luxS* of *B. longum* E18	this study
pBAD_LuxS_NCC_	Amp^R^, pBAD-based vector for arabinose-inducible expression of *luxS* of *B. longum* NCC2705	this study
pBAD_LuxS_S17_	Amp^R^, pBAD-based vector for arabinose-inducible expression of *luxS* of *B. bifidum* S17	this study
pBAD_LuxS_Vh_	Amp^R^, pBAD-based vector for arabinose-inducible expression of *luxS* of *V. harveyi* BB170	this study
pMgap	Spec^R^, vector for constitutive expression of proteins in bifidobacteria driven by the *gap* promoter (P*_gap_*) of *B. bifidum* S17	this study
pMgap_LuxS_NCC_	Spec^R^, vector for P*_gap_*-driven expression of *luxS* of *B. longum* NCC2705	this study
**Oligonucleotide**	**Sequence (5′ → 3′)** a	**Restriction Site**
VH1	GG ACTAGT ATGCCTTTATTAGACAGC	SpeI
VH2	CC ATCGAT TTAGTCGATGCGTAGCTC	ClaI
LF	GG ACTAGT ATGGCAGAAGAAACGGC	SpeI
LR	CC ATCGAT TCAGACGACCTTGCGCTC	ClaI
SLF	GG ACTAGT ATGGCAGAAGAAAAGCCG	SpeI
SLR	CC ATCGAT TCAGACGACCTTGCGCTC	ClaI
eLF	GGC CTCGAG ATGGCAGAAGAAAAGCCG	XhoI
eLR	CCC AAGCTT TCAGACGACCTTGCGCTC	HindIII

a recognition sequences for restriction enzymes are underlined.

### AI-2 like activity assay

AI-2 activity in culture supernatants was measured essentially as described elsewhere [9]. In brief, cell-free culture supernatants were prepared by centrifugation at 12,000×g for 5 min and subsequent filter-sterilization using membrane filters with a pore size of 0.2 µm. 20 µl of cell free supernatants or appropriate dilutions thereof were mixed with 180 µl of a 5,000-fold diluted overnight (o/N) culture of *V. harveyi* BB170 in individual wells of a black 96-well microtiter plate with flat transparent bottom (BRAND, Cat# 781611) and incubated at 28°C with shaking. Luminescence was measured every hour in an Infinite M200 multilabel plate reader (Tecan). AI-2 activity was quantified as relative luminescence units (RLU) at the time point when the negative control (AB medium instead of culture supernatants) produced the lowest amount of luminescence. Supernatants of an o/N culture of *V. harveyi* BB170 was used as positive control.

### Homologous and heterologous expression of luxS genes

For heterologous expression of *luxS* of *B. longum* NCC2705, *B. longum* E18, *B. bifidum* S17 or *V. harveyi* BB170 the respective genes were amplified from chromosomal DNA using specific primer pairs (VH1/VH2 for *V. harveyi*; LF/LR for *B. longum* NCC2705 and E18; SLF/SLR for *B. bifidum* S17; for sequences see Table 1). PCR amplification was performed using Phusion High Fidelity DNA Polymerase (Thermo Scientific). PCR products were inserted into pBAD [17], a vector for arabinose-inducible expression, immediately downstream of the P_BAD_ promoter in the correct open reading frame using the unique restriction sites for enzymes SpeI and ClaI. The resulting plasmids were introduced into *E. coli* DH5α by electroporation [18] and transformants were selected on LB agar with 100 µg/mL ampicillin. Positive colonies were checked for correct inserts by restriction analysis and sequencing.

For expression, strains were inoculated in LB medium and grown aerobically at 37°C on an orbital shaker. At an OD_600_ of 0.5, L-arabinose was added to the indicated final concentrations and growth was continued for further 4 hours. Cell free supernatants were collected for AI-2 activity assays and lysates of bacterial cells were analysed by sodium dodecyl sulphate polyacrylamide gel electrophoresis.

For the homologous expression, the *luxS* gene of *B. longum* NCC2705 was amplified using primers eLF/eLR (sequences see Table 1), and cloned into plasmid pMgap (Table 1) under control of the promoter of the *gap* gene of *B. bifidum* S17 between the unique restriction sites for enzymes XhoI and HindIII. The resulting plasmid pMgap_LuxS_NCC_ was introduced into *B. longum* NCC2705 by electroporation as described previously [17]. Transformants were selected on MRSc agar containing 100 µg/mL spectinomycin. Positive clones were confirmed by restriction analysis and sequencing of the plasmids.

### Biofilm assay

The ability of biofilm formation was assayed by classical crystal violet staining as described elsewhere [19]. Briefly, o/N cultures of bifidobacteria were diluted 1∶100 in fresh MRSc broth and transferred to 96-well microtiter plates (180 µl) with a hydrophilic surface (Sarstedt, Cat# 83.1835). 20 µL MRSc or supernatant of cultures of *B. longum* NCC2705 or derivatives were added. Plates were sealed and incubated at 37°C under anaerobic conditions for 48 h. Biofilms were stained with 20 µL of a 0.1% (w/v) crystal violet solution. Non-adherent bacteria were removed by washing three times using PBS. Crystal violet was released from biofilms by the addition of 200 µl of 100% ethanol and biofilm formation was quantified by measuring absorbance at 562 nm in a Infinite M200 multilabel plate reader (Tecan).

### Bioinformatic analysis

Amino acid sequences of the LuxS homologues of *Bifidobacterium sp.* were obtained from the Protein database at the National Center for Biotechnology Information and were searched for conserved domains using the web-based InterProScan 4 software of the European Bioinformatics Institute. Sequences were aligned using CLC Main Workbench 6 (CLC bio). The same software was used to calculate the LuxS phylogenetic tree with a neighbour joining algorithm. Bootstrap values were calculated with 1,000 replicates.

### Statistical analysis

All experiments were performed with at least three independent cultures per strain or condition and with technical replicates as indicated in the figure legends. Data was analysed by pair wise comparisons to the respective control using Student's *t*-test and *p* values <0.05 were considered significant.

## Results and Discussion

### Detection of LuxS homologues in bifidobacteria

*B. longum* NCC2705 represents the only strain of the genus *Bifidobacterium* for which a *luxS* homologue and corresponding protein have been reported so far [10], [11]. Interestingly, the LuxS protein of *B. longum* NCC2705 (LuxS_NCC_) appears to be hyperphosphorylated when bacteria are in their natural habitat within the gastrointestinal tract of the host [12] suggesting a role for LuxS under *in vivo* conditions. This prompted us to analyse the presence and function of *luxS* homologues in the genus *Bifidobacterium* in more detail. Putative *luxS* genes were found in all publically available *Bifidobacterium sp.* genomes (supplementary Table S1). The phylogenetic tree calculated with deduced LuxS amino acid sequences (supplementary Figure S1) is in good agreement with 16S rDNA-based phylogeny. This is in line with previous reports [10] and suggests that LuxS is evolutionary conserved with occasional horizontal transfer between distantly related clades. For example, the *luxS* gene of *B. longum* NCC2705 was shown to cluster with *luxS* genes of members of the *Firmicutes* of the genera *Lactobacillus*, *Lactococcus* and *Streptococcus* [10].

**Figure 1:**
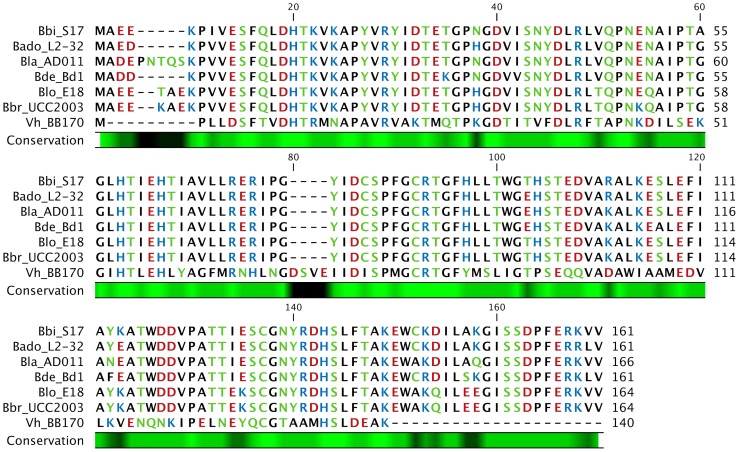
Alignment of the LuxS sequences of *B. longum* NCC2705, *B. longum* E18, *B. bifidum* S17, *B. breve* UCC2003, *B. adolescentis* L2-32, *B. lactis* AD011, *B. dentium* Bd1 and *V. harveyi* BB170. Amino acids are color-coded according to their polarity and the degree of conservation amongst the sequences is indicated below the alignment on a black-green scale with black indicating low and green high conservation.

All bifidobacterial LuxS homologues analysed consist of a conserved LuxS superfamily domain (data not shown) indicating a common function as a MTA/SAH nucleosidase. BLAST analysis of the LuxS protein of *B. bifidum* S17 (LuxS_S17_) revealed that the bifidobacterial LuxS sequences are >82% identical (data not shown). Alignment of a selection of bifidobacterial LuxS proteins illustrates the high conservation within the genus and homology to the LuxS of *V. harveyi* BB170 (LuxS_Vh_; Figure 1).

### AI-2-like activity in recombinant E. coli strains expressing bifidobacterial LuxS

**Figure 2:**
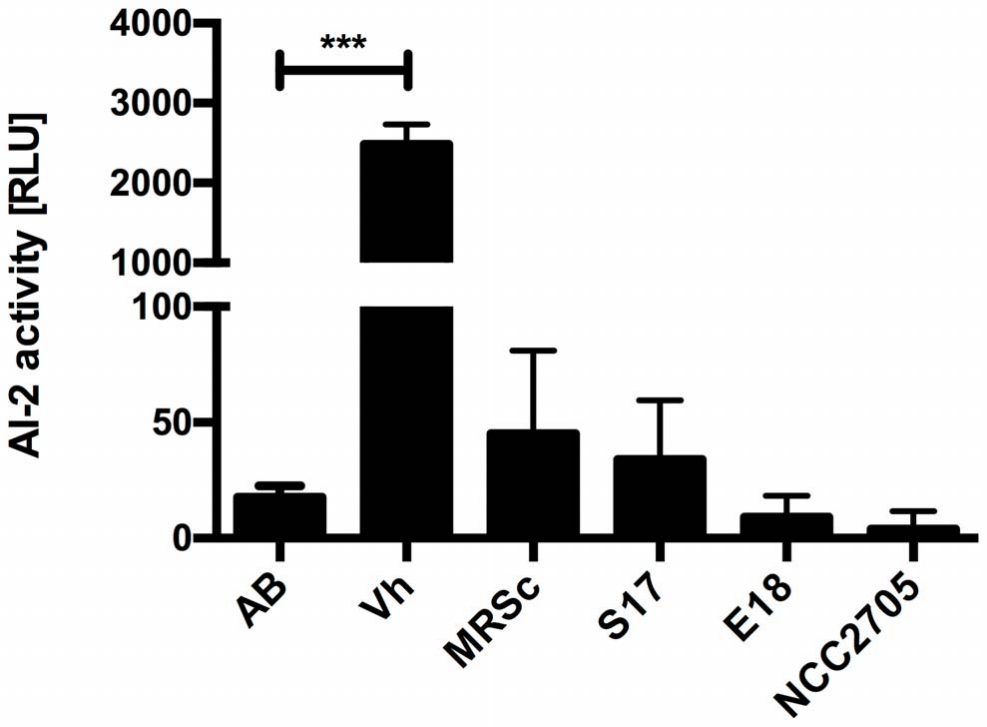
AI-2 activity in culture supernatants. *V. harveyi* BB170 (Vh), *B. bifidum* S17 (S17), *B. longum* E18 (E18) and *B. longum* NCC2705 (NCC2705) were grown in AB (Vh) or MRSc (bifidobacteria) medium to stationary growth phase. Sterile AB or MRSc medium was used as negative controls. Values are mean ± standard deviation (SD) of four replicates per condition and results of one representative of at least three independent experiments are shown. Data was analysed by pair-wise comparison to the respective medium controls (AB for Vh or MRSc for S17, E18 and NCC2705) using Student's *t*-test (***: *p*<0.001).

To check whether the bifidobacterial LuxS proteins are involved in AI-2 production, AI-2 activity was analysed in culture supernatants of three *Bifidobacterium* strains using the *V. harveyi* BB170 assay. Interestingly, we were unable to detect AI-2 activity in the supernatants of any of the tested strains of bifidobacteria grown in MRSc with the standard protocol (Figure 2). The assay itself appeared functional since the positive control, i.e. supernatant of *V. harveyi* BB170, yielded high levels of luminescence.

**Figure 3:**
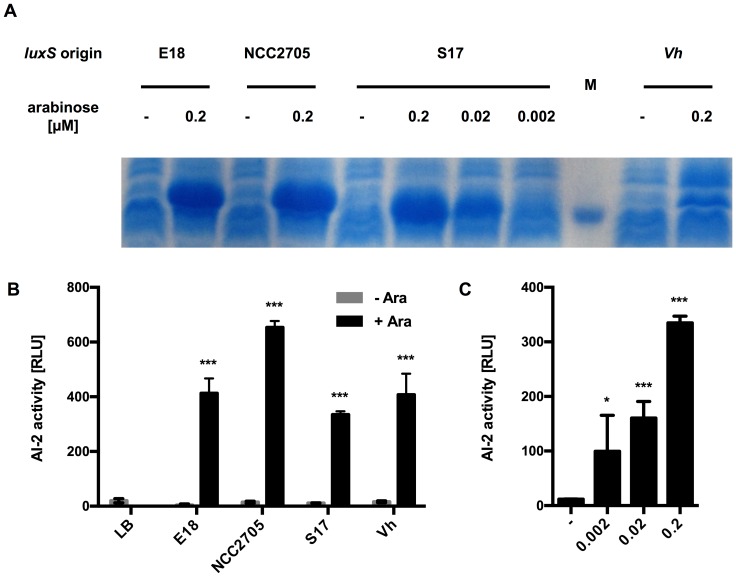
LuxS expression and AI-2 activity of recombinant *E. coli* strains. (A) Coomassie-stained SDS-PAGE of crude extracts of *E. coli* DH5α harbouring pBAD_LuxS_E18_ (E18), pBAD_LuxS_NCC_ (NCC2705) or pBAD_LuxS_S17_ (S17). Expression of *luxS* genes was induced for 4 h with arabinose at the indicated concentrations. Non-induced cultures (-) served as negative controls. The marker band represents a protein of 15 kDa. (B) AI-2 activity in supernatants of *E. coli* strains induced with 0.2 µM arabinose were analysed and compared to non-induced cultures. (C) AI-2 activity in culture supernatants of *E. coli* DH5α pBAD_LuxS_S17_ induced with different concentrations of arabinose. Results from one representative of at least three independent cultures for each strain are shown. Values in (B) and (C) are mean ± SD of four replicates per supernatant. Data was analysed by pair-wise comparison to the respective negative controls (LB in B; - in C) using Student's *t*-test (*: *p*<0.05; ***: *p*<0.001).

In order to assay the functionality of the LuxS proteins of the selected bifidobacteria, their *luxS* genes were cloned under control of the arabinose-inducible P_BAD_ promoter in pBAD and expressed in the AI-2 negative *E. coli* strain DH5α (Figure 3A). The *luxS* gene of *V. harveyi* BB170 served as a positive control. Following induction with arabinose, all recombinant *E. coli* strains expressed a protein of the expected size of about 17 kDa and expression was dose-dependent as shown for LuxS_S17_ (Figure 3A).

AI-2 activity could be detected in the culture supernatants of all recombinant strains after induction at levels comparable to those observed with LuxS_Vh_ and this AI-2 activity was absent in non-induced cultures (Figure 3B). Moreover, AI-2 activity in the supernatant of *E. coli* pBAD_LuxS_S17_ correlated with the arabinose concentration used for induction (Figure 3C). This clearly demonstrates that the cloned bifidobacterial *luxS* genes encode for functional MTA/SAH nucleosidases producing AI-2-like molecules.

### AI-2 activity of MRSc-grown bifidobacteria

Surprisingly, no AI-2 activity was detected for any of the tested bifidobacteria grown in MRSc to exponential (data not shown) or stationary growth phase when the AI-2 assay was performed using the standard protocol, i.e. 10% culture supernatant (Figure 2). Using this assay, AI-2 activity has been detected in supernatants of a wide range of bacteria [6], [8]. Moreover, the lack of AI-2 activity in culture supernatants of MRSc-grown bifidobacteria is not in agreement with the presence of AI-2 activity in the supernatants of recombinant *E. coli* DH5α strains expressing LuxS_NCC_, LuxS_S17_ and LuxS_E18_.

A number of factors including composition of the culture medium and the carbon source have been shown to have an impact on luminescence by *Vibrio sp.* [20]–[22]. Glucose present in the culture medium has been reported to inhibit the AI-2 signal of e.g. *Listeria monocytogenes* and several *Lactobacillus sp.* and catabolite repression was proposed as the mechanism of inhibition [23], [24]. Additionally, acidic pH was identified as another factor negatively impacting on AI-2-dependent luciferase reporter activity and thus neutralization of culture supernatants prior to AI-2 assays was suggested to improve detection [24], [25].

MRSc, i.e. the standard culture medium for bifidobacteria, contains 20 g/L glucose and the end products of the bifidobacterial metabolism on hexoses are mainly acetic and lactic acid. We thus performed growth experiments in MRSc and measured pH. All strains tested grew to comparable final OD_600_ and with comparable kinetics in MRSc (Figure S2). A marked acidification was observed even at early time points during growth and pH was close to 4 at 16 h after inoculation (Figure S2), i.e. the time when supernatants were collected for the experiments shown in Figure 2.

**Figure 4:**
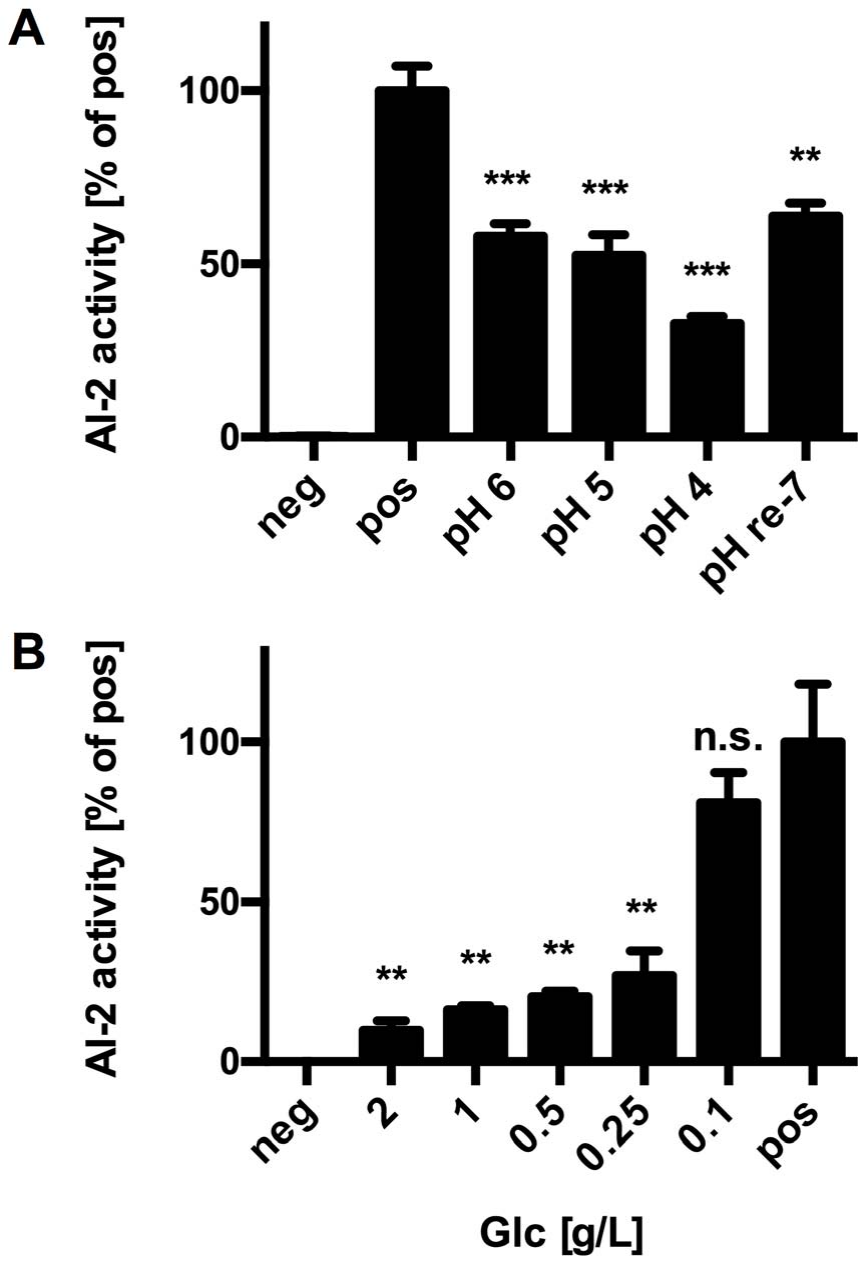
Inhibition of AI-2 activity by acidic pH and high glucose concentrations. AI-2 activity in supernatant of *V. harveyi* BB170 adjusted to the indicated pH (A) or spiked with different concentrations of glucose (Glc; B). Values are mean ± SD of one representative supernatant measured in three replicates and similar results were obtained with supernatants of three independent cultures. Data was analysed by pair-wise comparison to the respective positive controls (pos) using Student's *t*-test (**: *p*<0.01; ***: *p*<0.001).

To further test the hypothesis, that acidic pH and residual glucose are responsible for the lack of AI-2 activity in bifidobacterial culture supernatants, experiments were performed with supernatants of *V. harveyi* BB170 adjusted to different pH values before inoculation with the reporter strain. Acidic pH of the tested supernatants negatively affected detection and at pH 4, i.e. the pH observed in bifidobacterial supernatants, AI-2 activity was reduced to approx. 40% (Figure 4A). Re-neutralisation of *V. harveyi* BB170 supernatant previously acidified to pH 4 did not completely restore AI-2 activity. Thus, the inhibitory effect of acidic pH seems to be at least partially irreversible suggesting that the proposed neutralisation of culture supernatants [24], [25] might improve detection but does not allow for quantitative analysis as observed previously [23].

Similar to the pH experiments, *V. harveyi* BB170 supernatants were spiked with different concentrations of glucose prior to AI-2 assays. At 0.25 g/L of glucose, AI-2 activity was markedly inhibited to about 40% of the control (Figure 4B).

**Figure 5:**
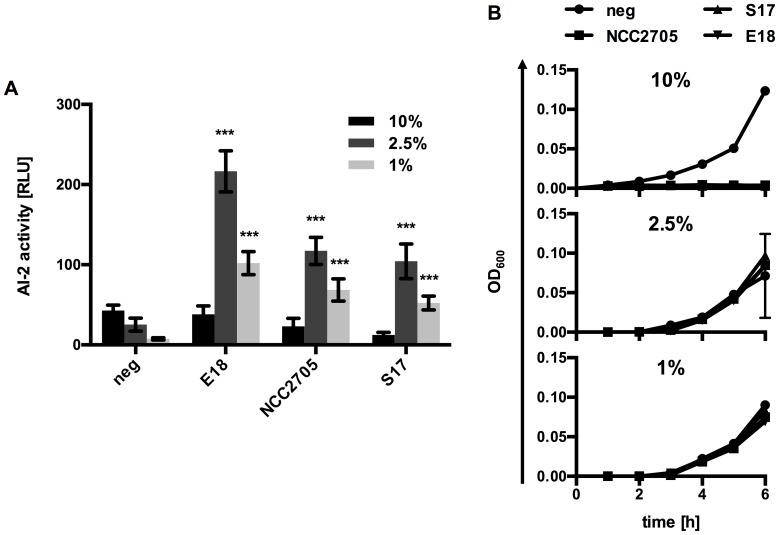
Improved detection of AI-2 activity in bifidobacterial culture supernatants. AI-2 activity (A) and growth of the reporter strain *V. harveyi* BB170 (B) using 1%, 2.5% or 10% culture supernatant of either *B. bifidum* S17 (S17), *B. longum* E18 (E18) or *B. longum* NCC2705 (NCC2705) grown in MRSc. Sterile MRSc was used as negative control (neg). Values are mean ± SD of one representative culture per strain measured in four replicates and similar results were obtained with supernatants of three independent cultures. Data in (A) was analysed by pair-wise comparison to the respective negative controls (neg) using Student's *t*-test (***: *p*<0.001).

In an attempt to improve detection of AI-2 activity in the culture supernatants of MRSc-grown bifidobacteria, further assays were performed using reduced volumes of culture supernatants. When 2.5% or 1% culture supernatants were used, AI-2 activity could be detected (Figure 5A) and the signal to noise ratio increased at lower concentrations due to a markedly reduced background in the negative controls, i.e. MRSc medium. These results clearly indicate that the tested strains produce considerable amounts of AI-2.

Also, growth of the reporter strain *V. harveyi* BB170 was inhibited by 10% supernatant but was indistinguishable from the respective negative controls at lower concentrations of supernatant (Figure 5B) providing yet another explanation for the lack of detectable AI-2 activity in bifidobacterial culture supernatants using the standard protocol. Whether growth inhibition of *V. harvey*i BB170 is exclusively related to low pH and residual glucose in the MRSc supernatants or whether other factors, e.g. antimicrobials produced by bifidobacteria, inhibit growth of the reporter strain remains to be investigated. For further AI-2 assays, 1% culture supernatants were used to minimize the inhibitory effect of low pH, residual glucose and other growth-inhibiting factors potentially produced by bifidobacteria.

### AI-2-like activity of bifidobacteria is LuxS-dependent and is linked to biofilm formation

**Figure 6:**
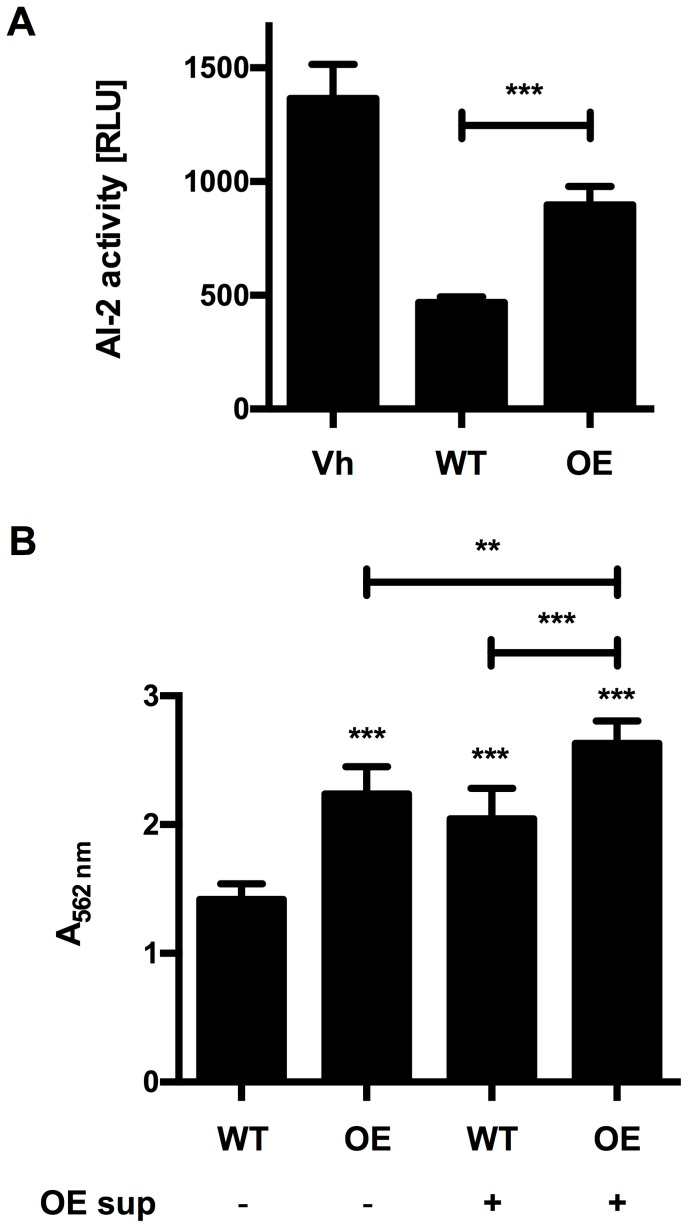
AI-2 activity and biofilm formation of B. longum NCC2705 can be increased by homologous over-expression of LuxS_NCC_. (A) AI-2 activity in the culture supernatant of *V. harveyi* BB170 (Vh), *B. longum* NCC2705 (WT) or its *luxS* overexpressing derivative *B. longum* NCC2705 pMgap_LuxS_NCC_ (OE). (B) Biofilm formation of WT or OE grown in MRSc with (+) or without (−) supplementation with 10% filter-sterilized culture supernatant of OE. Values are mean ± SD of one representative culture per strain measured in four (A) or eight (B) replicates and similar results were obtained with supernatants of three independent cultures. Data was analysed by pair-wise comparison to the wildtype controls (WT) or as indicated by the bars using Student's *t*-test (**: *p*<0.01; ***: *p*<0.001).

To further analyse whether AI-2 activity is linked to *luxS* expression in bifidobacteria, the *luxS* gene of *B. longum* NCC2705 was cloned under control of the *gap* promoter in a derivative of pMDY23 [26] for constitutive over-expression. A recombinant strain harbouring the resulting plasmid pMgap_LuxS_NCC_ showed about 2-fold higher AI-2 activity in cell culture supernatants compared to the vector control (Figure 6A).

Recent studies have indicated that in *Staphylococcus aureus* and lactobacilli LuxS has rather a metabolic role than being dedicated to production of a QS signal [25], [27], [28]. On the other hand, AI-2-dependent QS phenotypes have been demonstrated for a number of Gram positives even in the absence of the LuxPQ two component system or LsrB [5]. For example, LuxS and/or AI-2-like activity has been shown to affect biofilm formation of *L. rhamnosus* [25] and *L. monocytogenes* [29], [30].

To check if AI-2 activity also has functional relevance for bifidobacteria, biofilm formation of the LuxS over-expressing strain *B. longum* NCC2705 pMgap_LuxS_NCC_ was measured and compared to that of wildtype *B. longum* NCC2705. The wildtype strain exhibited biofilm formation on hydrophobic surfaces when grown in MRSc (Figure 6B). Over-expression of LuxS led to a marked increase in biofilm formation by approx. 50% (Figure 6B). Similarly, incubation of *B. longum* NCC2705 in MRSc supplemented with 10% culture supernatant of the overexpressing strain increased biofilm formation by 40%. Moreover, the overexpressing strain showed an increase in biofilm formation by 70% when grown in MRSc supplemented with 10% of its own culture supernatant.

## Conclusions

Our results demonstrate that all bifidobacteria harbour LuxS homologues, which are functional in all strains tested and result in the production of AI-2-like molecules. Moreover, the AI-2-like signal in the supernatant produced by LuxS plays a role in the formation of biofilms.

Bifidobacteria do not possess a LuxPQ two component system or a LsrB homologue ([5] and data not shown). However, LuxS of *B. longum* NCC2705 was shown to be hyperphosphorylated at serine and threonine residues specifically in the intestinal tract of the host [12]. Recent advances in proteomics and mass spectrometry have suggested that protein phosphorylation is common in bacteria and has a major impact on bacterial physiology [31], [32]. Thus, it is possible that the two isoforms of LuxS_NCC_ represent different activation states differing in their phosphorylation pattern. Further studies are required to investigate if LuxS is phosphorylated in response to AI-2 and whether this mechanism is present not only in NCC2705 but also other (bifido)bacteria. This would represent a novel mechanism by which the AI-2 signal is transduced to affect QS independent of a LuxPQ- or LsrB-type AI-2 receptor.

While AI-2-dependent QS phenotypes have been studied mostly in pathogenic microorganisms [3], AI-2-like molecules have been detected in the culture supernatants of commensal and potentially beneficial bacteria [25], [28]. In a range of pathogens AI-2-dependent QS was shown to regulate biofilm formation and adhesion to host cells thus promoting colonization and virulence [3], [8]. It seems only logical that sensing of and responding to self-produced AI-2 molecules (and those produced by other bacteria) might also play a role in the regulation of colonization factors and bacterial components involved in the effects of commensals or probiotics on the host. However, a direct impact of AI-2 molecules on the beneficial properties of a probiotic strain has not been demonstrated so far.

## Supporting Information

Figure S1 **Phylogenetic tree calculated with the amino acid sequences of the bifidobacterial LuxS homologues shown in Table S1.**
(PDF)

Figure S2 **Biomass (OD_600_) and pH of** ***B. longum*** **NCC2705,** ***B. longum*** **E18 and** ***B. bifidum*** **S17 during growth on MRSc medium under anaerobic conditions at 37°C.**
(PDF)

Table S1 **Bifidobacterial LuxS homologues of found in publically available genome databases with their corresponding locus tag and GI number.**
(PDF)
