# Supplementary material for: Bifidobacteria Exhibit LuxS-Dependent Autoinducer 2 Activity and Biofilm Formation
Source: PLoS One. 2014 Feb 5;9(2):e88260. doi: 10.1371/journal.pone.0088260 (PMC3914940; doi:10.1371/journal.pone.0088260)

Figure S2: Phylogenetic tree calculated with the amino acid sequences of the bifidobacterial LuxS homologues shown in Table S2.

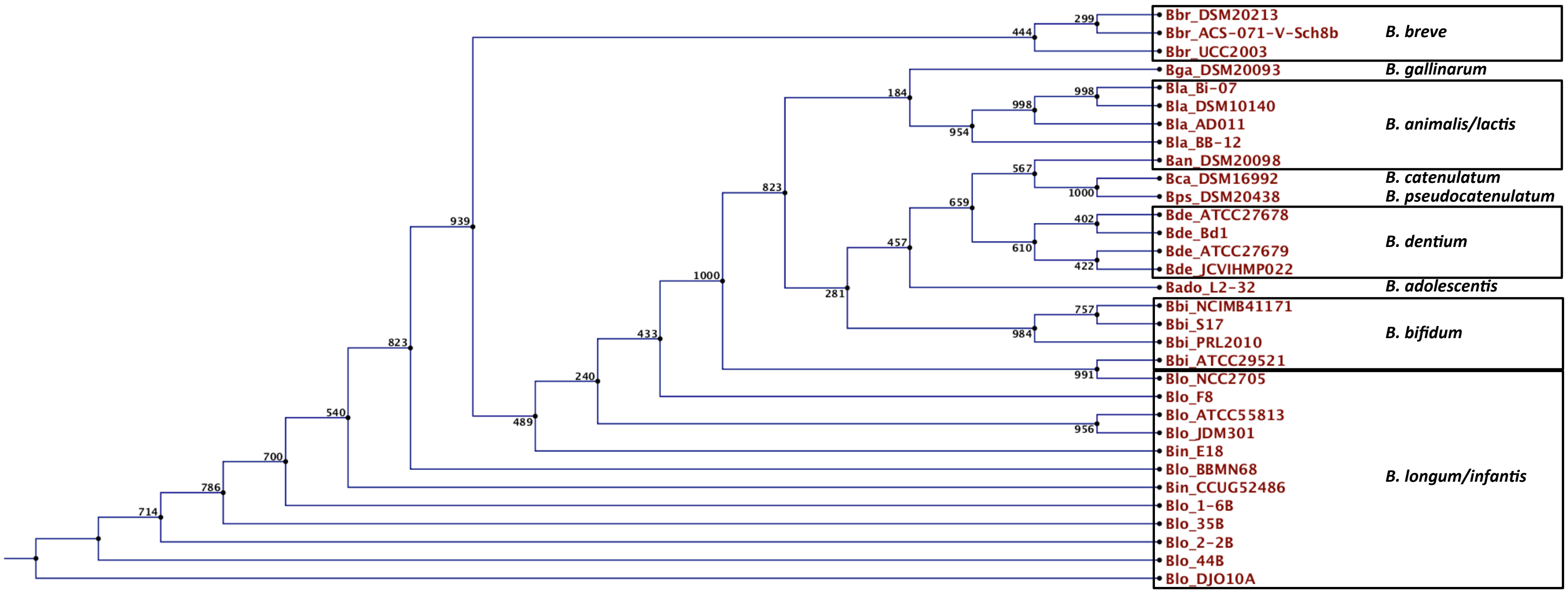

Supplement: Figure S1 — Phylogenetic tree calculated with the amino acid sequences of the bifidobacterial LuxS homologues shown in Table S1. (PDF) [file pone.0088260.s001.pdf]
