# Supplementary material for: Bifidobacteria Exhibit LuxS-Dependent Autoinducer 2 Activity and Biofilm Formation
Source: PLoS One. 2014 Feb 5;9(2):e88260. doi: 10.1371/journal.pone.0088260 (PMC3914940; doi:10.1371/journal.pone.0088260)

Figure S3: Biomass (OD_{600}) and pH of *B. longum* NCC2705, *B. longum* E18 and *B. bifidum* S17 during growth on MRSc medium under anaerobic conditions at 37 °C

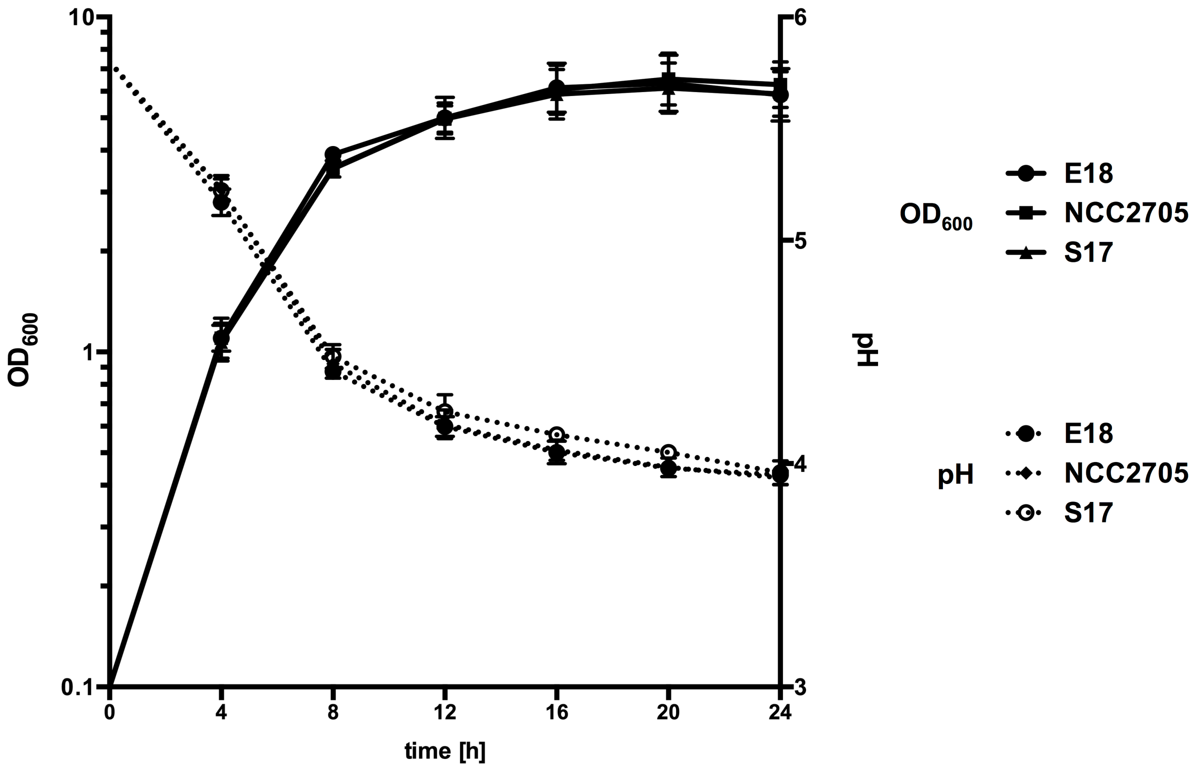

Supplement: Figure S2 — Biomass (OD600) and pH of B. longum NCC2705, B. longum E18 and B. bifidum S17 during growth on MRSc medium under anaerobic conditions at 37°C. (PDF) [file pone.0088260.s002.pdf]
