# Supplementary material for: Bifidobacteria Exhibit LuxS-Dependent Autoinducer 2 Activity and Biofilm Formation
Source: PLoS One. 2014 Feb 5;9(2):e88260. doi: 10.1371/journal.pone.0088260 (PMC3914940; doi:10.1371/journal.pone.0088260)
Supplement: Table S1 — Bifidobacterial LuxS homologues of found in publically available genome databases with their corresponding locus tag and GI number. (PDF) [file pone.0088260.s003.pdf]

Supplementary files

Table S1: Bifidobacterial LuxS homologues of found in publically available genome databases with their corresponding locus tag and GI number.

Strain	Locus tag	GI No.
B. adolescentis L2-32	BIFADO_00782	154084812
B. angulatum DSM 20098 (= JCM 7096)	BIFANG_03320	229784636
B. animalis subsp. *lactis* AD011	BLA_1115	219621246
B. animalis subsp. *lactis* BB-12	BIF_01888	384190872
B. animalis subsp. *lactis* Bi-07	W91_0606	386654775
B. animalis subsp. *lactis* DSM 10140	Balat_0582	240250585
B. breve UCC2003	Bbr_0541	476417675
B. bifidum PRL2010	BBPR_1341	310867022
B. bifidum ATCC 29521 (= JCM 1255)	unassigned	117574395
B. bifidum NCIMB 41171	BBNG_01295	313133130
B. bifidum S17	BBIF_1299	310287820
B. breve ACS-071-V-Sch8b	HMPREF9228_1355	333109577
B. breve DSM 20213 = JCM 1192	BIFBRE_03448	291381520
B. dentium ATCC 27678	BIFDEN_00080	171276628
B. dentium ATCC 27679	HMPREF0168_1301	304553073
B. dentium JCVIHMP022	HMPREF9003_0698	308220660
B. dentium Bd1	BDP_0675	283102232
B. catenulatum DSM 16992 (= JCM 1194)	BIFCAT_01757	212660103
B. pseudocatenulatum DSM 20438 (= JCM 1200)	BIFPSEUDO_02401	225158283
B. gallicum DSM 20093	BIFGAL_03594	270276714
B. longum subsp. *infantis* CCUG 52486	BLIG_00145	239514329
B. longum subsp. *longum* ATCC 55813	HMPREF0175_0753	227213310
B. longum subsp. *longum* BBMN68	unassigned	312133179
B. longum DJO10A	BLD_0910	189428208
B. longum subsp. *longum* 44B	HMPREF1312_0146	386417703
B. longum subsp. *longum* 2-2B	HMPREF1315_1080	386411300
B. longum subsp. *longum* 35B	HMPREF1314_1056	386408262
B. longum subsp. *longum* 1-6B	HMPREF1313_0335	386407207
B. longum NCC2705	BL1152	23326400
B. longum subsp. *longum* F8	BIL_13850	479136053
